# Lectures on Inflammation and Ulceration of the Cervix Uteri—In Answer to the Croonian Lectures of Dr. Charles West, on the Same Subject

**Published:** 1855-02

**Authors:** Henry Miller

**Affiliations:** Professor of Obstetric Medicine in the Medical Department of the University of Louisville


### MEDICINE AND SURGERY.

**February, 1855.** **NEW SERIES.** **Vol. III.—No. 2.**

### Art. I.—LECTURES ON INFLAMMATION AND ULCERATION OF THE CERVIX UTERI—IN ANSWER TO THE CROONIAN LECTURES OF DR. CHARLES WEST, ON THE SAME SUBJECT.

BY HENRY MILLER, M. D., PROFESSOR OF OBSTETRIC MEDICINE IN THE MEDICAL DEPARTMENT OF THE UNIVERSITY OF LOUISVILLE.

### LECTURE II.

The first lecture, which I had the pleasure to deliver in answer to the first of Dr. West's Croonian course on ulceration of the Os Uteri, was intended to counteract any unfavorable impression which his disparagement of the cervix, as a portion of the uterus, might make upon the mind of a candid inquirer and also to demur against a resort to *post-mortem* examination as wholly gratuitous. The contradictory and suicidal nature of the testimony, gathered from this source, was likewise pointed out.

Believing that his subsequent lectures are not less sophistical and therefore opposed to the simplicity of truth, I propose to follow him, step by step, and correct the aberrations to which he is, as we have seen, but too prone. In the exordium of his second lecture, Dr. West, with the transparent candor for which I have already given him proper credit, reminds Mr. President that the facts and considerations which he had the honor to submit in his first lecture, were not brought forward as conclusive but as elucidatory of the question; he nevertheless insinuates that they seemed to raise a presumption against rather than in favor of the opinion that inflammation of the cervix and ulceration of the orifice of the uterus are conditions of great pathological importance! In this he displays a degree of philosophical humility above all praise, for had he succeeded in proving, as undoubtedly he believed, that the neck is nothing and that it is very frequently beset with nothings, in the shape of superficial ulcerations, I submit that his work was accomplished and his warfare ended. What need of two more lectures to exult over a fallen foe? Its life ought at least to have been spared to grace the triumphal procession of his subsequent lectures.

Considering that the complex character of disease offers one great impediment to our thoroughly understanding it, Dr. WEST was induced to propose that we should, if possible, study:—

"As the *third point* in this enquiry, ulceration of the os uteri, under some condition in which it presents itself to our notice unconnected with other disease, and with such circumstances as to admit readily of our observing its characters, and noting its course and consequences."

Dr. WEST finds all these conditions for the study of ulceration, its symptoms and effects, its progress and consequences, uncomplicated by any other disease whatever, in cases of prolapsion of the uterus beyond the external parts, where moreover it can be easily inspected. Is prolapsus, then, not a disease? Supposing such a displacement to be not accompanied by inflammation or engorgement (which, however, is rare), is the lesion of situation nothing? It and its kindred affections, retroversion, anteversion, inversion, &c., have usually been regarded as such deviations from the normal state as to have found a place in Treatises on the Diseases of Females. So far from presenting a state of case in which ulceration of the os uteri can be abstractly studied, it does certainly seem to me that procidentia uteri greatly complicates the problem and makes it very difficult to determine what is to be attributed to the unnatural position of the organ and what to ulceration of its orifice.

In cases of complete prolapsion, the inverted vagina furnishes an investment to the misplaced organ, and its lining membrane gradually assumes the characters of ordinary integument, while the lips of the os uteri retain their original delicate structure and are, according to Dr. West, permanently in a state of abrasion or superficial ulceration. The inverted vagina is also implicated, and the ulcers upon it are apt to become deep and excavated, with raised and callous edges, exactly resembling chronic ulcers of the skin of other parts of the body.

And now for the study of these abrasions, which the, as he would persuade us, immaterial accident of procidentia has luckily thrown in our way, and we must take Dr. West for our guide, as such cases are by no means so common with us as he reports them to be in England:—

"The abrasions of the os, however, after weeks or months still retain much the same characters as they originally presented. They extend, indeed, at one time over a larger extent of surface than they occupy at another; but they very rarely increase in depth, or extend into the subjacent tissue. The ulcerated surface is denuded of epithelium; now and then it is partially covered by a thin layer of yellowish lymph, but usually it is of a rather vivid red color, and of a granular appearance. This granular character is generally more marked in proportion to the age of the ulceration; while in a few instances the granulations are distinct from each other, rather elongated in form, and look exactly like hypertrophied papillæ. A transparent albuminous secretion in general covers the ulcerated surface, and is sometimes poured out freely from it; but there is seldom any abundant discharge from the interior of the uterus, or even from the canal of the cervix."

Nor does the existence of prolapsus, complicated with such ulceration as he describes, incapacitate the uterus for the performance of all its functions, even the highest, provided it be retained in the cavity of the pelvis by some mechanical contrivance. Conception may readily take place and pregnancy and parturition be gone through with as formerly, with little or no additional suffering.

Of all the shifts and subterfuges resorted to by Dr. West to maintain his position, this, afforded by the procident uterus, appears to me the least tenable. It is manifest to the most superficial thinker that the abrasions of the os uteri and the deep ulcers of the vagina may be nothing more than the consequences of friction and the irritation necessarily caused by the dribbling of urine over the mucous surfaces. Abrasions and ulcers, produced by such purely mechanical causes, although perpetuated for an indefinite length of time, are never more than trivial local affections, and instead of increasing may be expected to diminish, as the parts become accustomed to such exposure. They are not, pathologically speaking, genuine phlegmasia and are incapable of giving rise to grave symptoms or of involving the deeper-seated tissues in the results of veritable inflammatory action. They may be good exemplars of Dr. WEST's idea of cervical inflammation and its sequences, but every practical observer must recognize a wide difference.

But allowing, for the nonce, that these urinous abrasions and exulcerations are models of the disease in controversy, we must not, in trying to estimate their value, lose sight of the comparative insensibility and diminished influence of the uterus, consequent on its exoteric position. While it is *in situ*, inflammation of the neck or incipient prolapsus may affect the bladder and rectum, teasing them by traction or involving them in inflammation or a high degree of irritation. Hence, dysuria and tenesmus are among the most common symptoms of cervical inflammation. It is easy to conceive what an amount of morbid influence may thus be radiated from the pelvic viscera, deranging the functions of other organs in sympathy with them. But let the uterus achieve its fall, drawing the vagina along with itself and converting it into a hernial sac, with an almost cutaneous envelope, into which the ovaria and tubes, the bladder and part of the rectum and some convolutions of the small intestine are received,—it and they soon become accomodated to their novel situation, all strain is taken off from its supports that had been hitherto kept tense by their resistance, and a notable mitigation of the most distressing symptoms ensues. Dr. BLUNDELL does but repeat what must be familiar to all, when he says: "In procidentia of the uterus, it is remarkable that the health of the patient often suffers very little. Indeed it has been often observed, with truth, that the general health is often much worse in those cases in which there is a mere relaxation, than in those cases of procidentia which we have just been considering; in which the vagina and uterus lie forth under view."*

From this consideration, it is manifest that even supposing the

* Principles and Practice of Obstetric Medicine, London, p. 700.

abrasions of the procident uterus to be of the same nature as those of the organ *in situ*, they are comparatively innocuous, and that, therefore, any conclusions drawn from one are not logically applicable to the other, unless it can be shown that like effects are to be expected from like causes under dissimilar conditions.

We have at length arrived at the fourth and most important part of this inquiry, which is to ascertain "what clinical observation generally teaches us concerning ulceration of the os uteri, its course, its symptoms, and its importance." In approaching this question, as Dr. West declares we are bound to do with no conscious bias of the mind in one or the other direction, three different probabilities suggest themselves to us, of which any one may be correct.

"1st. Ulceration of the os uteri may be the cause of all the symptoms of uterine disease which have been attributed to it; and consequently it may be of no less importance to remove it when present, than to ascertain the fact of its existence.

"2nd. Though not in itself the cause of the symptoms, or at least of the greater part of them, it may yet be the concomitant of certain forms of uterine disease; of the state and progress of which its extent and degree may be a trustworthy index. In this case, though of small importance as far as therapeutical proceedings are concerned, it may yet be of great semiological value.

"3rd. Neither the one or the other of these suppositions may be correct; but either the ulceration may exist alone, giving rise in that case to few symptoms, or to none at all; or it may, in other instances, complicate different uterine ailments, though not an index of their state, nor varying with their changes."

Considering that, in the opinion of some writers, so large a proportion as eighty-one per cent. of all women presenting symptoms of uterine ailment, are suffering from inflammatory disease of the tissue or canal of the cervix, and 70·4 per cent. likewise from ulceration of the os uteri, Dr. West thinks the inquiry may be very briefly disposed of,—for the evidence in support of such a view must, he alleges, be overwhelming, the symptoms of ulceration of the os uteri must be characteristic, either from their peculiarity or severity, or from both together, and must differ, in important respects, from such as attend upon those uterine ailments which are not associated with that condition. Here is a string of propositions, stated in the language of Dr. West himself, upon which his subsequent reasoning turns and from which his eventual conclusion is deduced, and yet these propositions or premises are not only not proved but wholly gratuitous and unfounded.

When, in investigating the pathological state of the womb, we discover, by tactile and ocular proof, that there is inflammation of the cervix and are not able to detect any other lesion of the organ or its appendages, why should we refuse to allow this morbid condition of the cervix to be the *causa symptomatorum* unless its vouchers are *overwhelming?* Shall inflammation of the *corpus uteri* or no appreciable lesion of any kind be entitled to rank as a cause of uterine symptoms upon evidence less irresistible? It is easy to discover, in such rigorous exactions of the cervix, the influence of the bias deprecated by Dr. WEST, but which has, nevertheless, so possessed him that it cannot be exorcised.

His second assumption demands that the symptoms of ulceration of the os uteri shall be characteristic and differ materially from those of all other uterine ailments,—a hard requisition and one with which it may not be possible for the symptoms of any uterine disease to comply. There is not a disease of the uterus, whether it be imflammation of the body or neck, polypus, fibrous tumor or even cancer, prolapsus, inversion, anteversion or retroversion, which is always accompanied with characteristic symptoms, such as serve to diagnose it without physical examination. Dr. SIMPSON, the renowned Professor of Midwifery in the University of Edinburgh, in his "Contributions to the Pathology and Treatment of Diseases of the Uterus,"* lays it down as his first proposition that, "*The general and local functional symptoms of disease of the uterus are such as enable us to localize, without enabling us to specialize, the exact affection of the organ.*"

He illustrates and establishes the truth of this proposition, which does but embody the experience of all men and all time, by referring to the dissimilar effects of pregnancy, in different cases, and to the different phenomena of uterine disease, excited by the same specific affection of the organ, while, on the contrary, " he same specific phenomena frequently result from affections of the organ, that are entirely at variance with each other, in their pathological character, in their course, and in their treatment."

* London and Edinburgh Monthly Journal.

To instance some of these diseases. Scirrhous degeneration of the cervix uteri sometimes gives rise, at an early period of its progress, to severe pains in the uterine region and marked constitutional derangement. In other cases, which have come under my own observation, it pursues its course without pain and with such slight disorder of the uterine functions, including, as Dr. West would say, the highest, as to escape the notice of patient and physician, until its destructive ravages are well nigh completed. "While," as Prof. Simpson remarks, "we thus not unfrequently find the most malignant organic diseases of the uterus more or less latent or masked in their symptoms, we have, on the other hand, sometimes the most severe local and constitutional symptoms of uterine disease developed in instances of slight and remediable organic affections of the part, as in cases of simple ulceration and granular inflammation of the cervix; and these symptoms may be all present in their most aggravated forms for months and even for years, when the local examination and final result show us that there is no organic disease whatever as in cases of "irritable uterus" or hysteralgia. Indeed, in some females, we have all these symptoms strongly but temporarily excited at every recurrence of the catamenial discharge, in connection merely with that congestive and increased vital activity of the organ which accompanies its natural menstrual secretion."

There is a certain group of sufficiently well-defined symptoms, such as *pain in the sacral, pubic or iliac regions*, mostly dull but occasionally acute and paroxysmal, like parturient pains; *heat or burning* in these same regions; *sense of fullness and weight* within the pelvis; *bearing down; hemorrhagic and leucorrheal discharges; deranged menstruation; painful coition; and frequently recurring abortion*,—which point plainly enough to the uterus as the seat of disease of some kind, but none of them nor all of them, in any combination, infallibly indicates the special morbid affection, which may only be revealed by physical exploration.

But although the terms of its admission into the nosological nomenclature are hard and unreasonable, it may safely be affirmed that inflammation and ulceration of the cervix can approximate more nearly to them than any other uterine affection, its symptoms being better marked, and sometimes, indeed, quite pathognomonic, as will be presently shown.

From these considerations, it undeniably follows that to require inflammation of the cervix to exhibit, as its credentials, peculiar and sharply-defined symptoms, such as are not observed in any other uterine disease, or submit to be expunged from the record of this class of maladies, is an infringement of its rights, against which those charged with protecting them are bound to protest.

We are justified, by every principle of sound reasoning, in maintaining that if other lesions may derange the uterine functions and affect the general health, so may it, and that, if no other lesion can be detected, after the most sedulous examination, to ignore it or estimate it as nothing is a violation alike of sound philosophy and common sense.

When we are led by suspicious symptoms to investigate the condition of the uterus, it is often found that the neck is in a state of chronic inflammation, and no other disease whatever can be discovered, after the most careful and thorough examination. It stands revealed by the speculum in all the vividness and isolation of an ophthalmia and in connection with it some of the symptoms of the uterine category are developed. Notwithstanding that the symptoms themselves may be insufficient to point out this precise local disease, they do, nevertheless, harmonize with it and are such as may be more rationally attributed to it than to any other cause or to no cause at all. To illustrate this remark and at the same time to impress you with the pathological importance of such an affection, though seemingly small, I will offer an admirable description of one form of the malady by the pen of a writer, who had evidently often seen it and diligently studied it. The special form of the affection is chronic inflammation of the *mucous membrane lining the interior of the neck*, and the writer is M. MELIER, in a memoir entitled "*Considerations Pratiques sur le Traitement des Maladies de la Matrice.** Inflammation thus seated is regarded by M. MELIER as a veritable catarrh of the part, extending in some cases into the body of the uterus, and is of very common occurrence. The influence which it exercises

*Memoires de l' Académie Royale de Medecines, 1833, Tome Deuxième. p. 330.

over the development of other maladies and its ulterior consequences have not, he thinks, been justly appreciated. It may easily be discovered with the speculum, but eludes an investigation by the touch alone. Thus examined, the orifice is perceived to be more or less red, the mucous membrane extending into its cavity appears tumid; thick, viscid, gluey, white mucus, more or less colored and sometimes sanguinolent, is seen flowing or rather adhering, and obstructs the cervical canal, from which it can scarcely be removed by a mop. The orifice itself may be observed in two different states: it may be extremely contracted or very patulous and infundibuliform, from the swelling of its borders. The neck is sometimes tumefied, uneven and indurated; in other cases, it preserves its normal consistence and volume. Melier adds that he knows not how far the malady is limited to the neck, but it is probable that most frequently it extends to the body of the uterus. The patient complains habitually of a dull, deep-seated pain, sometimes of acute suffering, heat and pruritus behind the pubes, which she indicates as the seat of the malady. Changing their ordinary character, these pains may become more violent and resemble those of labor, and after a longer or shorter time, their sudden cessation is announced by a more abundant discharge than is usually present. The menses, more or less painful, are glairy from the admixture of mucus: coition is painful.

Furthermore, the cervical inflammation is represented by M. Melier as being very chronic, and in its course, in a longer or shorter time, a new pain is complained of in the inguinal or ovarian regions, indicating a tendency to propagation of disease to one or both ovaria. These organs may become involved and then a tumor is discovered in one or both sides, and the pain becomes more acute and persistent. The neck is primarily affected and for a time suffers alone. Subsequently and by the sympathy of continuity, the ovaria participate in its sufferings and take on inflammatory action, which may result in degeneration of their tissues or hypertrophic enlargement. Interrogate the patients, says M. Melier, and go back to the very beginning: they will tell you that the malady commenced in the hypogastrium, behind the pubes, and extended consecutively to the ilia. This succession—

these two stages—were well-marked in a patient whom I am now attending with my learned friend, Dr. S. C. ROCHE. Attacked with a catarrh of the uterine neck, she complained only of this *for one year*, at the expiration of which, the right iliac region was affected, and then there was notable enlargement of the ovary of that side. M. MELIER affirms that he has observed the same march, the same succession in many other cases, and, he doubts not, that much of ovarian disease might be prevented by the detection and cure of the cervical malady. This form of cervical inflammation might, with more propriety, be denominated *Mélier's Malady* than the whole class to which it belongs can be called *the Miller disease*—an honor, though not so intended, which some have conferred on myself, and my only regret is that I am not worthy of it.

MELIER's malady is often overlooked. Too many are apt to confine their examination to the exterior of the neck and, if they find no disease in the mucous membrane that covers it, hastily conclude that the cervix is in a healthy state, although its *lining* membrane is deeply inflamed. Dr. WEST, with his contemptuous notions of the neck, and looking only for "trifling abrasions" upon the cervical exterior, has doubtless often failed to detect comparatively occult disease. For my own part, I can truly declare that inflammation of the lining membrane has often come under my notice and I can bear testimony to the accuracy of M. MELIER's description of it. Whether ovarian inflammation and morbid degeneration are as often produced by it, as he thinks, I cannot undertake to decide. I have several times observed the co-existence of the two affections, but my opportunities for exact observation have not allowed me to determine their order of succession. This, however, I know, in common with all who have any clinical acquaintance with it, that ovarian pain is among the most common symptoms of cervical inflammation, and there can be no good reason to doubt that a cause, capable of disturbing these organs to that degree, may eventually kindle in them inflammatory action. Such a propagation of inflammation, it may be added, is not more strange than orchitis produced by gonorrhœal inflammation of the urethra.

Here, then, is a disease as well made out as any other in the nosological catalogue, which approximates as nearly as possible to the sublime requisition of Dr. West, and we may fairly demand its recognition as a distinct uterine disease, unless it can be shown that some other affection of the organ has been better studied and is better understood. Let Dr. West designate, if he can, that affection.

Having pointed out the defects in his premises, which vitiate his reasoning, we are prepared to follow Dr. West in the comparison which he institutes between the two grand classes into which he is pleased to divide all uterine diseases, namely, those in which there is, and those in which there is not, ulceration of the mouth of the womb. The object of the comparison is stated to be to ascertain "whether sterility is more frequent, whether the rate of fecundity is lower, and whether abortion occurs oftener in one class of cases than in the other? Whether menstrual disorder is more common, more severe, or different in kind; whether leucorrhœa is more abundant, or furnished from a different source; or whether pain is less tolerable when the os uteri is ulcerated, than when that condition is absent? And lastly, whether similar or different causes produce the uterine affections in the two classes of cases; whether the duration of illness is the same; whether the structural alterations of the womb are alike or diverse?" It is not difficult to detect the radical vice that permeates this statement or to prognosticate the vicious conclusion to which it must needs conduct the learned Croonian. If, in the particulars enumerated, there shall be found no marked difference between the two classes of cases, but, on the contrary, that a great resemblance obtains, so that women, laboring under uterine disorders, may have the same symptoms and results, whether ulceration of the os uteri be absent or present, then, in his own language, "it may be inferred with equal certainty that ulceration of the womb can neither be regarded as a general cause of uterine disease, nor as a trustworthy index of its progress; but that it is a pathological condition of secondary moment, and this even though there be still some difficulty in assigning to it in every instance its proper value." Seeing that there is, as has been shown, remarkable identity in the symptoms of diverse diseases of the womb, the conclusion which I would draw from the premises and which is unquestionably the legitimate one, is, that different causes, and among them inflammation of the cervix, may give rise to identical symptoms and consequences.

Having opened his cause, Dr. WEST proceeds to spread before us the materials from which he hopes to make some approach to a satisfactory answer to the question he had propounded concerning the *role* of ulceration of the os uteri. These materials are derived from 1226 cases at the Middlesex and St. Bartholomew's Hospital. In only 268 of them did the symptoms appear to him to justify the use of the speculum, and in 125 of these the os uteri was found to be the seat of ulceration, while in the remaining 143, it showed no sign of that condition. He very properly declines using any except the cases examined with the speculum, for the solution of the question before us. These furnish him with the grounds on which the different conclusions rest, and before we proceed with him to work out, or rather unwork, these conclusions, it may not be uninteresting to offer a few words of commentary on the data which were at his disposal. It strikes me as strange that out of the total number of 1226 females, with symptoms of uterine ailment, Dr. WEST should have thought it not necessary to a correct diagnosis to employ the speculum in more than 268 cases. Unless their complaints were exceedingly trivial, I am persuaded that useful light might have been thrown upon them, by a resort to that much abused but invaluable instrument. Then again, that he should have found only 125, less than half the number examined specularly, affected with ulceration, is to myself conclusive proof that either he is sadly deficient in practical sagacity, or women in England are different from their sex in this country,—for, without laying claim to any special tact in this particular, I am confident that I have found inflammation in a far greater proportion of cases, where I had reason to expect it.

Again; it may be instructive to compare Dr. WEST's researches with those of Dr. BENNET, who is connected with another public institution in London, and who, in my opinion, is justly entitled to rank among the highest and most reliable authorities on this subject. Dr. BENNET, as every body knows, affirms the greater frequency and importance of inflammation and ulceration of the cervix in comparison with the body of the uterus, and avers that, during the last few years, he has kept a careful register of all the cases of uterine disease which he has treated at the Western General Dispensary, with which he is connected as physician-accoucheur. This Dispensary is, as he informs us, one of the largest institutions of the kind in London, nearly ten thousand patients being annually treated by its medical officers. His own patients consist of those who present uterine symptoms, and on analyzing their cases, 300 in number, the details of which were all scrupulously recorded, he finds that 243 were suffering from decided inflammatory disease of the cervix or its cavity and that in 222 ulceration was present, which is in the proportion of one in every 1·57 for inflammation, and one in every 1·78 for ulceration. The analysis of the cases, he further states, which he has seen and attended in private practice during the last three years,—amounting to nearly 300,—leads to precisely the same conclusion. Dr. Bennet considers the local inflammation the real cause of the morbid symptoms, notwithstanding the great diversity in the *apparent* nature of the disease. "Some patients," says he, "complained of leucorrhea, some of dysmenorrhea, some of irregular menstruation, some of flooding, some of backache, some of bearing-down and prolapsus, and some merely of debility and anemia. The true nature of the case had to be sifted out—as generally occurs; what was only a *symptom* being considered the disease."

During the last fifteen years, a large number of females have come under my treatment for sexual diseases,—some of them residing in the city but many more from abroad,—and although I have kept no regular record of their cases, I am very sure that in a large majority of them, inflammation with or without ulceration of the cervix uteri was the special malady and the point of departure of most of their ailments. I do not mean to affirm that it always presents itself in an isolated state, for it is sometimes associated with inflammation of the mucous membrane of the body of the organ, whether by gradual extension or simultaneous occurrence, I cannot undertake to decide. I am, also, well aware that inflammation may be confined to the body, or at least, exist in that portion of the uterus in a more decided degree than in the neck.

Thus much premised, we can give to Dr. West the attention he craves to the various points, already referred to as likely to elucidate the question of the influence of ulceration of the os uteri in the production of uterine disease or in occasioning functional disorder of the generative system.

1. *Parallel between ulcerative and non-ulcerative uterine diseases in their influence upon reproduction.* To learn whether uterine ailments in general or ulceration of the os uteri in particular exert any pernicious influence over the generative function, Dr. WEST collected the statistics of 980 healthy married women of different ages, who were attended in their confinements by pupils of Saint Bartholomew's Hospital, and the like number, married above one year, who applied during the childbearing period of life on account of any ailment of the uterine system,—125 of whom, being examined with the speculum, were found to be unaffected by ulceration and 117 of the number being affected by it. The information thus collected he embodies in tabular form, from which it appears that there were 4·17 children and ·7 abortions to each marriage of healthy women; 2·7 children and ·47 abortions to each woman with uterine symptoms; 3·3 children and 1·3 abortions to each woman with uterine symptoms but without ulceration; and 3·5 children ·89 abortions to each woman having uterine symptoms with ulceration.

This table discloses what we should have expected, namely, that uterine disease is unfavorable to fecundity; it discloses, also, what we should not have expected, viz: that ulcerative disease is not so unfavorable to propagation as anomalous and anonymous complaints of the womb. The difference between the two classes of cases, viz: the ulcerative and the non-ulcerative, is, as Dr. WEST confesses, but small, and the number of cases from which the table is constructed are too few to justify any such inference; "but I do think," he says, "that we are warranted in concluding that ulceration of the os uteri does not interfere with the performance of the most important function of the generative system in any peculiar manner, or to a greater degree than many other uterine ailments." My own conclusion would be that although ulceration of the os uteri is not an insurmountable barrier to fecundity, yet *it diminishes fecundity nearly as much as all other diseases besides*,—the figures being 3·5 to 3·3, while its proportion of abortions is as ·89 to 1·3, of all that occur in females laboring under uterine complaints. This conclusion rests on the basis, already sought to be established for it, namely, that if no other disease could be detected, it alone is to be held responsible for the pathological results.

The observations of Dr. West, thus tabulated and paraded as of mighty statistical moment, are, however, so deficient in details as to be well nigh valueless. We do not learn anything from him except that these cases were accompanied with *uterine symptoms*, and ulceration was found in a certain number but absent in the remainder; that each class bore a certain number of children and aborted a certain number of times! As to the ulcerative cases, we are not informed how long the local lesion had existed or whether it was discovered only once or oftener by specular examination. For aught that appears to the contrary, the ulceration might have supervened upon the last parturition or abortion and consequently might have had no agency either in promoting or thwarting fecundity.

From researches so sterile in available information it is refreshing to turn to those of Mr. Whitehead, of Manchester, who has, by indefatigable labor in an almost boundless field and amid scenes calculated to repel most men, collected and systematically arranged a body of accurately observed facts, bearing on this question, from which we feel safe in drawing definite and positive conclusions. The results of these laborious investigations he has given us in an unpretending volume, entitled "Causes and Treatment of Sterility and Abortion,"—a monument to his memory more enduring than even "written rocks." His cases occurred among the patients of the Manchester Lying-in Hospital,—an institution peculiar, as he tells us, in having no beds, its very numerous patients being attended at their own houses or hovels, and consisting of the humbler order of females, mostly factory operatives. From thousands of these he collected the history of the reproductive period (*twenty years*) of their lives; thousands of them were, moreover, examined with the speculum, and thus a mass of information was gathered, unequalled on this subject and scarcely surpassed on any other.

Our present inquiry is concerned only with the *causes* of abortion, with some allusion merely to its treatment, so far as it confirms the verity of the causes assigned. Mr. WHITEHEAD questioned two thousand women on their admission as patients in the Manchester Lying-in Hospital, as to their previous history in reference especially to their unsuccessful pregnancies, and the causes assigned by them and their medical attendants for the misfortune in each instance. Of this number, seven hundred and forty-seven had already aborted once at least; to some, the event had occurred several times. The sum of their abortions was twelve hundred and twenty-two. The following are the causes to which they had been attributed:—

| | |
|---|---|
| "Inward weakness," impaired state of the health generally, and acute disease, | 911 |
| Accidents, mental perturbations, &c. | 221 |
| No assignable cause, | 90 |
| | 1222 |

By the term "inward weakness" and its equivalent, "waste," which is usually employed by the uneducated poor in England and also in this country, is always meant *leucorrhea*, *fluor albus*, or *whites*. The form of the affection in which the discharge consists in part of pus, the common people of Manchester denominate the "yellows," expressive of its color, which they rightly regard as a more serious malady. Mr. WHITEHEAD was frequently struck, in the course of his inquiries, with the commonness of leucorrheal discharges previous to abortion, in connection with a well marked train of local and constitutional disturbances, and on resorting to the speculum in such cases, the source of the discharge appeared to be plainly revealed;—"disease of the lower part of the uterus being found to exist in almost every instance." That this lesion of structure constitutes the true pathological seat of leucorrhea and of all its associated phenomena, as well as a very frequent cause of disastrous events during pregnancy, is, he thinks, proved by the beneficial effects of remedies directed to the uterine affection, such as cauterization with the nitrate of silver, &c., as in cases uncomplicated by pregnancy. With the improvement of the local affection, the discharge diminishes, the patient becomes more comfortable, being relieved of most of the symptoms that had distressed her before previous abortions, and she may go the to full time and give birth to a healthy child.

Mr. Whitehead subjected to careful investigation three hundred and seventy-eight cases of abortion, principally with a view to obtain a correct statistical average of its prevailing causes, and the result is embodied in the subjoined table.

*Causes of, and conditions associated with Abortion, in Three Hundred and Seventy-eight Cases.*

| Accidental agencies. | Placenta prœvia. | Constipation of the bowels. | Retroversion of the uterus. | Incurable disease. | Vascular congestion. | Disease of the lower part of the uterus. | Obscure causes. |
|---|---|---|---|---|---|---|---|
| 44 | 8 | 3 | 3 | 1 | 15 | 275 | 29 |

Upon these divers causes of abortion he offers judicious practical remarks, illustrated and enforced by a recital of cases; but we are at present interested only with *disease of the lower part of the uterus* (the cervix), considered as a cause of abortion, which figures largely in this table, claiming seventy-three per cent., as its proportion. These were, with very few exceptions, examined with the speculum, either before the abortion took place or in three or four weeks after, and in every case, disease of the lower or of the internal part of the uterus, and in a few instances of the vagina was found to exist. Some who had the disease in a severe form submitted to treatment and were cured; others disappeared after being prescribed for two or three times. One hundred and forty-one of these patients have a second, some a third time presented themselves for treatment, being again pregnant and affected precisely as they were on previous occasions. As to the success of remedial measures directed chiefly to the local affection, in preventing abortion, in such as submitted, a second or third time, to treatment, we are told,—"In fifteen of those who have been a second time treated, the issue has terminated unfavorably. Fifty-four have already arrived at the full period of the process; of whom three were delivered of still-born children, and in fifty-one the child was born alive and in health in each case. In the remainder, the treatment has been so for successful as to lead to a confident hope that the issue will be favorable."

The discovery of ulceration of the cervix uteri in pregnant females is attributed by Dr. BENNET to M. BOYS DE LOURY, one of the physicians of Saint Lazarre, a hospital prison in Paris, by whom it was recognized as a frequent cause of abortion. This opinion was adopted and confirmed by Dr. BENNET prior to the date of Mr. WHITEHEAD'S publication. To the former, indeed, the profession in Great Britain and this country are indebted for their knowledge of the subject. Dr. BENNET describes, however, but one variety of the affection,—a modification of the simple inflammatory ulceration of the non-gravid state. The hypertrophy, increased vascularity and softening of the womb during pregnancy changes the aspect of this form of ulceration in a very striking manner,—its surface becoming granular and even fungous, while there is also a more abundant purulent secretion. Mr. WHITEHEAD is entitled to the credit of pursuing the investigation and greatly extending our knowledge of diseases of the uterus, especially of the cervix, in the pregnant state, which may eventuate in abortion, and I will offer a brief synopsis of his classification of them. Under the general expression of *disease of the lower part of the uterus*, he includes the following lesions:—

1. *Inflammation and superficial erosion*, implicating one or both lips of the os uteri, and more or less of the external and internal cervix.

2. *Varicose ulceration*, commonly occupying the back part of the anterior lip, sometimes confined to the posterior, and occasionally implicating both. It gives rise to hemorrhages obeying, more or less perfectly, the menstrual periods and to purulent discharges in the intervals.

3. *Œdema of the Cervix.*

4. *Fissured ulceration* of one or both commissures, of the anterior or posterior lip, or implicating all these parts at the same time.

5. *Induration of the cervix*, with or without abrasion of surface.

6. *Endo-uteritis*, or inflammation of the lining membrane of the uterus, affecting the body as well as the neck, and sometimes accompanied with induration of the cervix or erosion of one or both lips of the os uteri.

7. *Follicular ulceration.*

8. *Gonorrheal inflammation*, affecting the lips and adjacent cervix, and especially liable to spread to the lining membrane of the entire organ.

9. *Syphilitic disease*, in its primary, secondary and tertiary stages.

10. *Prolapsus uteri*, which owes most commonly its existence to disease of the lower part of the uterus, as the primary exciting condition.

Not only has abortion been traced by Mr. WHITEHEAD to these several lesions, mostly of the cervix, but he even assigns to each its causal proportion and indicates the period of pregnancy when each is most apt to occasion abortion. Thus, inflammatory ulceration was the cause in 26 in every 100, and the event happened between the middle of the sixth and the middle of the ninth month of pregnancy. Varicose ulceration induced abortion in 6 or 8 out of every 100, and it operated during the latter two or three months. Œdema of the cervix, acting likewise in advanced pregnancy, prevailed in about 1 in every 25 or 30 cases. Fissured ulceration was found to exist in 20 to 24 out of every 100 cases, and may cause abortion from the fourth to the middle of the seventh month of uterogestation. Endo-uteritis is a common cause of abortion during the early months. It may implicate the cavity of the cervix only, or involve the whole lining membrane of the womb.

The argument does not need greater amplification:—what has been declared is sufficient to warrant the affirmation that if any pathological doctrine can be established by clinical observation, then has Mr. WHITEHEAD proved, beyond all reasonable doubt, that inflammatory disease of the cervix uteri is a frequent cause of abortion. If this be not the irresistible conclusion from his premises, then is all clinical experience futile and all ratiocination a vain delusion.

Let us inquire now, very briefly, whether cervical disease can be considered a cause of sterility and thus affect fecundity by preventing conception, while abortion acts by bringing to naught its product.

Sterility may doubtless be attributed to a variety of causes, some of which, such as lesions, or malformations of the fallopian tubes and ovaria, it may not be possible to discover during life. But there can be no doubt that, in a certain proportion of cases, inflammation of the cervix uteri constitutes the only assignable cause, the efficacy of which, as a barrier to conception, is proved by fecundation taking place subsequent to the subdual of the inflammation. I must not be understood to assert that inflammation of the cervix is necessarily and in every instance a cause of sterility, for many exceptions have come to my personal knowledge and doubtless the malady often exists antecedent to the pregnancy which it destroys by abortion. What may be affirmed, nevertheless, without the fear of successful contradiction, is the co-existence of such inflammation and sterility. the removal of the inflammation being followed by cure of the sterility, in too many instances to admit of the belief that they were merely coincident.

When sterility results from this cause, it is probable that the inflammation affects the lining membrane of the cervix and not unfrequently also that of the body,—the former being the malady described by MELIER and denominated *cervicitis* by WHITEHEAD; the latter, *endo-uteritis* of the last named author. When the affection is restricted to the exterior of the neck, it is not so apt to interfere with fecundity, although it may, as we have seen, blight its fruit and occasion abortion. The reason of the difference, in this respect, between external and internal inflammation of the neck is probably found in the obstruction of its canal by glairy mucus in the latter, offering an impediment to the intromission of spermatozoa, whose access to the ovule is known to be essential to fecundation. Be this as it will, you may observe that all the cases of sterility detailed by WHITEHEAD were accompanied with *endo-uteritis*, and some of the patients, who had undergone a tedious matrimonial probation, became fecund after being cured of their malady.

2. *Parallel between the two classes of cases in respect to their influence upon menstruation.* Are manifestations of menstrual disorder alike or diverse in ulcerative and non-ulcerative cases? There is, as Dr. WEST alleges, a general correspondence between them, and he gives a tabulated statement, comprising a gross total of one hundred and thirty-eight cases of anomalous uterine disease withour ulceration, and one hundred and twenty of ulceration, showing the manner in which the menstrual discharge was affected. I need not go into the details; it is sufficient to say that the difference between the two classes is not great—menstruation being natural or suppressed, irregular or scanty, painful or profuse in both, in nearly the same per cent. It is only remarkable that in those cases where there was ulceration there was somewhat greater activity of the sexual function. "In them, pregnancy or lactation was more frequent; scanty, irregular, suppressed, or painful menstrual was rarer; while excessive or over-frequent menstruation occurred with greater frequency." From which we are to conclude, I suppose, that ulceration only quickens the uterus in the performance of its functions! There is, I think, some inaccuracy in the observation of these cases; at all events, painful menstruation is more frequently connected with inflammation than is here exhibited. But admitting the truthfulness of his observations, still, on the principle which I have endeavored to establish, in all the cases of deranged menstruation in which no other cause could be assigned, the ulceration is fairly entitled to claim the paternity, and these make its progeny as numerous as that of all other causes united.

3. *Parallel between the two classes in their relation to leucorrheal discharges.* The object of the comparison is to determine whether such discharges are more frequent, more profuse, or furnished from a different source where ulceration exists, than in cases where it is absent. These particulars are exhibited, as usual, in tabular form; nearly an equal number of cases of leucorrhea with and without ulceration are contrasted, from which it is to be inferred that leucorrhea is nearly as often met with and may be equally profuse, where there is no ulcerative disease whatever. The source of the discharge was determined in eighty of the non-ulcerative, and eighty-five of the ulcerative cases, viz:—in fifty-four of the former, it was from the uterus, in nineteen from the vagina, in seven from both—80; while in fifty-three of the latter the discharge was from the uterus, in six from the vagina, in eight from both, also, in twelve, in an appreciable degree, from ulceration, in only six from ulceration alone—85.

By the discharge being from the uterus, Dr. West evidently means from the body of the organ, for in remarking on opinions contrary to his own, he says: "The discharge is supposed to be furnished either from the ulcerated surface itself, or from the inflamed and irritated canal of the cervix." This table, if it is to be received, develops the most astounding of all the disclosures of our lecturer, seeing that leucorrheal discharges have their source in the body of the uterus, whether there be ulceration of the os or not; in twelve cases, truly, the oral ulceration helped to swell the tide, but in only six was it the *fons et origo!* I am free to confess that, if it can be established by accurate observation that leucorrhea is independent of disease of the cervix, to the extent indicated by this table, the controversy is ended and Dr. WEST has exploded a pernicious error. The cervix is, in my opinion, the predominant and almost exclusive seat of leucorrhea, and to this portion of the uterus are nearly all local remedies for the malady addressed, which would be nugatory, of course, could it be proved that it arises from a higher source. Oppugnant to our doctrine and practice as are these statistics of leucorrhea and decided as is the vantage gained by them, if credited, Dr. WEST submits, for the present, only a few modest comments and leaves them to make their own impression upon the reader. The strong current, thus put in motion, only sinks, however, Arethusa-like, to reappear in the third lecture, where I shall endeavor to stem it.

4. *Parallel between the two classes in respect to the accompanying pains.* Comparison extended to another symptom, seldom absent in uterine affections, namely, pain, shows, as Dr. WEST thinks, a close correspondence between the two classes of cases. In both, the seat of pain is various; it may be in the uterus, in the back, the pubic or iliac region, or in two of these regions at the same time. A table turns up again, exhibiting one hundred and forty-three cases in which there was no ulceration and one hundred and twenty-five in which ulceration existed, and the seat of pain is specially indicated. All that is conceded to the ulcerative cases is, that the pain accompanying them is usually more generally diffused over the whole pelvic region. Pain is not to be regarded as characteristic of ulceration, either on account of its intensity or seat, and it is, every way, too unimportant in this discussion to merit the notice bestowed on it by Dr. WEST. Let it be conceded that any disease of the uterus may be attended with pain.

5. *Parallel between the two classes of cases in reference to the appreciable organic condition of the uterus.* This, as well as the other points of the inquiry, is sought to be illustrated by a tabular statement, setting forth the state of the womb, so far as it could be ascertained, in the ulcerative and non-ulcerative cases. The table (No. VIII.) comprises one hundred and thirty-nine cases of non-ulcerative and one hundred and twenty of ulcerative disease; the uterus (meaning the body) was apparently healthy in twenty-nine of the former and thirty-six of the latter; not healthy in some respect or other in one hundred and ten of the former and eighty-four of the latter. The deviations from a healthy state, indicated in the table, consisted in misplacements, enlargements, or indurations of the body or neck, affecting one portion only or both, at the same time. It would be uninteresting to go into the details, nor is it at all necessary. It will be sufficient merely to indicate their bearing on the question at issue. Dr. West thinks that this table exemplifies the same general correspondence between the two classes of cases; he admits, however, that enlargement of the body of the uterus and enlargement or induration of the cervix existed much more frequently in connection with ulceration of the os uteri than independently of that condition. Still he maintains that cases of enlargement and induration, unconnected with ulceration, are sufficiently numerous to repel the assumption that the latter is the cause of the former. "Moreover," says he, "in two-thirds of the cases where ulceration was absent, and in one-third of those in which it was present, the enlargement was confined to the body of the womb —a fact easily reconcileable with the belief that that part of the organ, as it is of the greatest physiological importance, so is also the more frequent seat of the gravest pathological processes, or, at any rate, their most usual point of departure."

That the body of the womb may be enlarged by other causes than ulceration of the orifice, I have no wish to deny. There is no reason why that portion of the organ may not be the primitive and exclusive seat of disease; at the same time, it cannot, I think, be doubted that inflammation may be propagated from the neck to the body, as described by Meleir, and there is good reason for the belief that, in all cases wherein both portions of the womb are implicated, the usual point of departure of morbid action is, contrary to Dr. WEST's opinion, the cervix instead of the body. In support of this view, it may be observed that the neck is far more frequently diseased than the body,—this table itself showing that while the body alone was enlarged in twenty cases of non-ulcerative, and twelve of ulcerative disease—32, the cervix alone was enlarged or indurated or both in thirty-four non-ulcerative, and twenty three ulcerative—57, which is nearly in the proportion of two to one, and yet falls short, I suspect, of the real preponderance of cervical over corporal affections of the womb. Now, let it be granted, as it must, that inflammation may spread in either direction along the continuous mucous lining of the uterus, and it necessarily follows that when both portions of the organ are found to be alike involved, the probability is at least as two to one that the morbid action commenced in the cervix.

"It also seems questionable, from the data which this table furnishes, whether induration of the os or cervix uteri is so generally dependent on ulceration of the os uteri as has been asserted, since it was present in forty per cent. of the cases where no ulceration existed. Besides, if such a connection as that of cause and effect subsisted between ulceration of the os uteri and induration of its cervix, or even if there were any necessary relation of degree between them, we ought to find the most extensive ulceration coexist with the greatest hypertrophy and most considerable induration; while slight ulceration of the os, and an otherwise healthy state of the cervix, might be expected to be usually found together. Facts, however, do not bear out this opinion."

Here follow two tables (IX. and X.) the first showing the different *seats and comparative frequency* of different forms of ulceration, and the second, the *degree*, marked "slight," "moderate," and "extensive," in which they existed. These tables possess no special interest and are of no importance to any one except Dr. WEST himself, who produces them merely to subserve a particular purpose, namely, to show

"1st. That in 25 out of the 46 cases in which the ulceration is stated to have been slight, more or less considerable induration or enlargement of the lips or neck of the womb was present.

2nd. That in 9 out of 16 cases in which the ulceration was stated to have been considerable, there was no induration nor enlargement either of the cervix or os uteri.

The argument is, I readily grant, conclusive against *ulceration*

being the cause of enlargement or induration either of the neck or body of the womb; but it has no relevance and is utterly powerless when applied to *inflammation*, which, as already shown in my first lecture, is really the disease in controversy. Ulceration, enlargement, and induration are all sequences of inflammation and there is no more reason to ascribe enlargement to ulceration than ulceration to enlargement, nor has any writer displayed such pathological imbecility, as is here imputed. Dr. WEST cannot be ignorant of the doctrines he criticises and his perpetual resort to a quibble,—substituting ulceration for inflammation, and making it the butt of his animadversions,—completely strips him of the cloak of fairness, which he affects to wear and is at such pains to display to the best advantage. These several lesions being only sequences of inflammation, we should expect that when one exists in an unusual degree, the others may be in abeyance. When, for instance, inflammatory action expends its whole force upon the mucous membrane, extensive ulceration with no apparent enlargement may be the consequence; but should the subjacent tissue become implicated, evinced by enlargement from deep-seated congestion, the result may be effusion of coagulable lymph and permanent induration, with little or no superficial ulceration. Such enlargement and induration of the cervix is a very common pathological condition, and whenever it is met with we may safely attribute it to inflammation. whether other evidence of that pathological state happen to be present or not. Ulceration may also persist after the subsidence of active inflammation, serving to complicate the results of that morbid process and being itself only an effect of their common cause, inflammation.

